# Knowledge of obstetric fistula and its associated factors among women of reproductive age in Northwestern Ethiopia: a community-based cross-sectional study

**DOI:** 10.1186/s12905-022-02001-8

**Published:** 2022-11-23

**Authors:** Meskerem Tsega Dejen, Tesfaye Assebe Yadeta, Getnet Gedefaw Azeze, Asmamaw Demis Bizuneh, Getahun Tiruye, Agumasie Semahegn

**Affiliations:** 1 Department of Midwifery, College of Medicine and Health Sciences, Dilla University, Dilla, Ethiopia; 2 School of Nursing and Midwifery, College of Health and Medical Sciences, Haramaya University, Harar, Ethiopia; 3 Department of Midwifery, College of Medicine and Health Sciences, Injibara University, Injibara, Ethiopia; 4 School of Nursing, College of Health Sciences, Woldia University, Woldia, Ethiopia; 5 Department of Population, Family and Reproductive Health, School of Public Health, University of Ghana, Accra, Ghana

**Keywords:** Women’s knowledge, Associated factors, Obstetric fistula, Northwestern, Ethiopia

## Abstract

**Background:**

Obstetric fistula has been a major maternal health challenges in low and middle-income countries, especially in Ethiopia, due to high child marriage and poor access to healthcare. Obstetric fistula is common among teenage mothers that results in a vast social, economic and cultural sequel. In Ethiopia, there is a paucity of research evidence on women’s knowledge about obstetric fistula. Therefore, this study aimed to assess women’s knowledge about obstetric fistula and its associated factors at Banja District, Northwestern Ethiopia.

**Methods:**

A community-based cross-sectional study design was conducted from 1st -21th July 2021. Systematic sampling method was used to recruit 784 women in the reproductive age from six rural and one urban sub-districts. Two days of training was given to research assistants regarding the objective of the study, inclusion and exclusion criteria, checking completeness and ways of protecting confidentiality. Data were collected using face-to-face interview method. Collected data were entered into EpiData and exported into SPSS version 24 for cleaning and analysis. Descriptive statistics, binary and multivariable logistic regression analysis were performed to identify associated factors. Adjusted odds ratio (AOR) at 95% CI with p < 0.05 was used to declare significant association with women’s knowledge of obstetric fistula.

**Results:**

A total of 773 women gave a complete response with response rate of 98.6% (773/784). One-third (36.4%; 95%CI: 32.9-39.7%) had good knowledge about obstetric fistula. Women who had completed primary education (AOR:3.47, 95%CI:2.01–5.98), secondary and above (AOR:3.30, 95%CI:1.88–5.80), being a student (AOR: 6.78, 95%CI:3.88–11.86), get counseling about obstetric fistula (AOR:6.22, 95%CI: 3.78–10.24), participated in pregnant women’s discussion forum (AOR:3.36, 95%CI: 1.99–5.66), had antenatal care follow-up (AOR: 2.40, 95% CI: 1.39–4.13), being an urban resident (AOR: 3.19, 95% CI: 1.33–7.66), and having access to Television/Radio (AOR:1.68, 95%CI:1.10–2.60) were significantly associated with women’s good knowledge about obstetric fistula.

**Conclusion:**

Women’s knowledge about obstetric fistula is unacceptably low. Therefore, concerned stakeholders should enhance awareness creation programs, strengthen antenatal care, counselling and women’s discussion forum that could substantially optimize women’s knowledge about obstetric fistula and its risk factors of obstetric fistula.

## Background

Globally, for every maternal death, an additional 20–30 women develop a serious pregnancy-related complication. Of these severe maternal morbidities, obstetric fistula is one of the most common devastating conditions [1]. An estimated 2 million women in developing countries are living with untreated obstetrical fistulas [2, 3, 4]. Of these, an estimated incidence of 30,000–130,000 obstetric fistula occurs in sub-Saharan Africa every year, which accounts more than 60% of overall burden [4, 5]. Fistula is an abnormal hole that can be exist in any part of the body and that results abnormal leakage between body parts, and obstetric fistula is a hole between the vagina and bladder, and/or between the vagina and rectum, which results in continuous leakage of urine or stool into the vaginal vault [6]. It is predominantly caused by injury during childbirth, resulting in an abnormal opening between the vagina and the bladder, either vesicovaginal fistula (VVF) or rectum (rectovaginal fistula (RVF) [1].

Obstetric fistula which is largely caused by prolonged and obstructed labor, is a good reflection of weak health system that failed to provide accessible, timely, and appropriate intrapartum care [6, 7], in developing countries where access to obstetric care is limited [8]. Among all maternal morbidities, obstetric fistula is considered the most devastating adversely affects both the physical and mental health of the women [9]. The women with fistula may have continuous leakage of urine, faces, or both that results in highly debilitating condition often end up with stigma and discrimination against the victim women. The physical and psychological sufferings adversely affect the quality of women’s lives in such a catastrophic way that they are sometimes described as dead women walking [10, 11].

Available evidences have shown that, 37.2% of women had poor knowledge, and 56.6% misconceptions about the causes/risk factors for obstetric fistula in Ghana [12]. Likewise, in Uganda, majority of women and few men had an awareness about obstetric fistula, but there is high misconception about the cause, clinical manifestations and prevention. Conception at an early age, delaying access in medical care, delivery by a traditional birth attendant and delivery by instrumental delivery are some of the risk factors [13]. One-in -three (36.4%) of women in Burkina Faso had good knowledge about obstetrical fistula and its prevention methods [14].

In Ethiopia, more than 110, 000 women have suffered from obstetric fistula, yielding the lifetime risk of experiencing obstetric fistula to be 1060 per 100,000 women [15]. Of these, only 2000 (2%) women get treatment in the last 3 years. These data imply that if no new cases occur, and with the current rate, it will take at least 55 years to treat the existing patients in Ethiopia [10]. Ending obstetric fistula is one of the critical measures to achieve the third Sustainable Development goal (SDGs) by 2030[16]. As result, the Ethiopian government devised and implemented several strategies such as reducing teenage pregnancies, improving access to obstetric care, creating awareness in the community about obstetric fistula complications, and instituting treatment modalities to prevent and control obstetric fistula [17, 18]. Yet, there have been between 3,300 and 3,750 new cases of obstetric fistula each year [17].

The burden of untreated obstetric fistula in 2016 was found to be high in the Amhara region of Ethiopia with 230 cases per 100,000 women of childbearing age [19]. Thus, lack of awareness among communities about the risk factors, prevention methods, and healthcare for obstetric fistula is the main barrier in minimizing complications, and improve timely treatment-seeking behavior [15]. Despite this effect, the knowledge level of the childbearing women on obstetric fistula and its associated factors has been remains a challenge in Ethiopia, especially in the Amhara region where the burden is unacceptably high [19]. There was paucity of evidence on women’s knowledge about obstetric fistula and its associated factors in the study area. Therefore, the main aim of this study was to assess women’s knowledge level about obstetric fistula and its associated factors at Banja District, Northwestern Ethiopia.

## Methods

### Study design, and setting

A community-based cross-sectional study was conducted at Banja District, Awi Zone, Amhara Regional State, Northwestern Ethiopia from 1st -21st July 2021. Banja District is one of the districts in Awi zone located 447 km away from Addis Ababa, and 120 km away from Bahir Dar in Northwestern Ethiopia. According to the Zonal Health Department report (2020), Banja District had a total of 100,836 population, of these, women (15-49years) account for 23,777 (23.6%). The district, comprised of 25 rural and 2 urban sub-districts/kebeles (*smallest administrative unit in Ethiopia*). Banja district has 6 functional health centers, 25 satellite health posts, 3 private medium clinics, and 2 private drug stores and one general hospital [20].

## Eligibility for participation

Women of the reproductive age group (15–49 years) who had been living in Banja District for at least six months at their respective sub-districts, and registered by health extension program (had family folders) were eligible for this study. Nevertheless, women who were severely ill and unable to give a response during the data collection period were excused from the study.

## Sampling methods

**Fig. 1:**
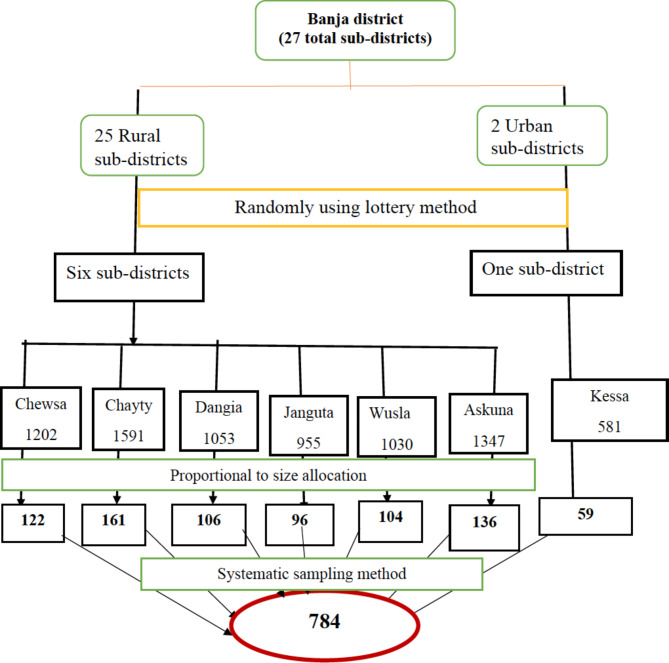
Schematic presentation of the sampling procedure obstetric fistula knowledge study in Banja District, Northwestern Ethiopia, July 2021

A single population proportion formula was used to determine the sample size using parameters; the knowledge of obstetric fistula among women 36.4% [21], 5% margin of error, 95% significance level and considering a design effect of two and 10% potential non-response compensation. The final sample size for this study was 784 women in the reproductive age. Six rural and one urban sub-districts were randomly selected out of 27 total sub-districts in Banja District. A total of 7,759 women in the reproductive age at households’ level was identified as eligible from registered family folder at respective satellite health posts of selected sub-districts. A sampling frame was constructed for selected sub-districts using the women’s list from family folder which was regularly updated by health extension workers in collaborative with sub-districts administrative bodies. The family folder comprised of household number (unique ID), sociodemographic characteristics of each household member, and vital events. Hereafter, the calculated sample size was proportionally allocated to the identified eligible households at each sub-district. Eventually, systematic sampling was used to select participants based on a sampling interval of (k = 10). In case more than one woman in a given household were identified, one woman was randomly selected using the lottery method. The sampling procedure is schematically presented using flow diagram (Fig. 1).

## Data collection method

Data were collected using face-to-face interviewer-administered pretested structured questionnaire which were adapted through review of relevant literatures [12, 21–27]. The questionnaire has consisted of socio-demographic characteristics, obstetrics-related characteristics, and knowledge-related characteristics. The questioner contains 65-item, and internal consistency of the questionnaire was estimated using Cronbach’s alpha, and found to be good (> 0.70). The English language questionnaire was translated into two local dialects (Amharic and “Awigna” by language experts and back-translated to English to check its consistency. Trained fifteen data collectors and three supervisors who can speak and write local dialects, both Amharic and “Awigna” languages were used to collect the data through house-to-house survey. The face-to-face interview was held approximately from 45 to 60 min.

## Quality control measures

To ensure data quality, three-day training was provided to data collector and supervisors about the objectives of the study and the data collection techniques. A pre-test was done outside of the study area (non-selected sub-districts) on 5% of the sample size (n = 40) to test skill of data collectors, content and sequence of question in the tool, sensitive nature of the tool, and time for interview at Akayta sub-district of Banja district. Then, correction and modification of the instrument were undertaken accordingly. Supportive supervision was offered to data collectors by trained supervisors and principal investigator. Then supervisors checked the completeness of the collected data collected on daily basis. In addition, double data entry had done to minimize data entry errors and to ensure the quality of the findings.

## Outcome variable measurement

Obstetrical fistula is an abnormal opening between a woman’s vagina and bladder and/or rectum, that results in the continuous involuntary leakage of either urine or faeces into the vaginal vault [28, 29]. Regarding knowledge of obstetric fistula: women’s comprehensive knowledge about obstetric fistula was measured using ten questions with 30 items that mainly comprised of ever heard of obstetric fistula, know the type of obstetric fistula, cause/risk factors, sign and symptom, treatment and prevention of obstetric fistula. The responses for each of the items was scored as “1 = correct answer” and “0 = wrong answers.” Each item was then summed up and the mean score was computed which was (0.36). Finally, women’s knowledge ≥ 0.36 score was categorized as ‘good knowledge about obstetric fistula’, and those women who scored below 0.36 were categorized as ‘poor knowledge of obstetric fistula’.

## Data processing and statistical analysis

Collected data were entered into EpiData version 4.2 and exported into SPSS Version 25 for cleaning and analysis. Descriptive statistics were applied to compute frequency, proportion, mean, and standard deviation. Wealth index had calculated using principal component analysis (PCA), and categorized it to 5 level of wealth (poorest, poor, medium rich and richest). Binary logistic regression analysis was carried out to check which explanatory variables have association with the outcome variable (i.e., women’s knowledge about obstetric fistula). To control for possible confounding factors, variables with P-value of ≤ 0.25 in the bivariate analysis were fitted in the final model using multiple logistic regression analysis. The goodness of fit was tested by Hosmer-Lemeshow statistic and Omnibus tests [30]. The adjusted odds ratio (AOR) at 95% CI with p-value < 0.05 was used to declare significant association with women’s knowledge about obstetric fistula.

## Results

### Sociodemographic characteristics of the study participants

In this study, 773) women of reproductive age were participated, yielding an overall response rate of 98.6% (773/784). The mean age of the women was 33.03 (± 9.61) years. Two-third (66.4%) of women and 79.3% of husbands were farmers. The majority (94.7%) of women were Orthodox Christianity religion followers, and 69.3% of women were married (Table 1).

**Table 1:** Sociodemographic characteristics of the participants in Banja District, Northwestern Ethiopia, July 2021(n = 773)

Variables	Category	n	%
Women’s age (Years)	15–19	107	13.8
	20–24	66	8.5
	25–29	109	14.1
	30–34	118	15.3
	35–39	145	18.8
	40–44	120	15.5
	45–49	108	14.0
Current marital status	Married	536	69.3
	Divorced	54	7.0
	Widowed	39	5.1
	Single	144	18.6
Religion	Orthodox	732	94.7
	Muslim	19	2.5
	Protestant	22	2.8
Womens education status	No formal education	405	52.4
	Read and write	111	14.4
	Primary education	123	15.9
	Secondary education	111	14.3
	College and above	23	3.0
Women’s occupation status	Housewife	51	6.6
	Government employee	22	2.8
	Private employee	48	6.2
	Farmer	513	66.4
	Student	139	18.0
Husband educational status (n = 536)	No formal education	284	53.0
	Read and write	121	22.6
	Primary education	66	12.3
	Secondary education	35	6.5
	College and above	30	5.6
Husbands’ occupation (n = 536)	Farmer	425	79.3
	Government employee	28	5.2
	Private employee	52	9.7
	Daily labourer	19	3.6
	Student	12	2.2
Residence	Urban	58	7.5
	Rural	715	92.5
Wealth index	Poorest	158	20.4
	Poor	152	19.7
	Medium	70	9.1
	Rich	279	36.1
	Richest	114	14.7

## Obstetrics related characteristics

The median age at first marriage was 16 with IQR 5 with a minimum of 10 and a maximum of 26 years. The median age at first pregnancy was 18 with IQR 4 with a minimum of 13 and a maximum of 30 years. The median age at first childbirth was 19 with IQR 4 with a minimum of 14 and a maximum of 31 years. Of 773 participants, 42.3% and 73.0% women were grand multigravidas and multiparous respectively. Women who had history of abortion and stillbirths were 16.7% and 9.2% respectively. More than three-fourth (78.1%) of women gave birth at health institutions, and two-third of the participants described that lack of transportation was the main reason for giving birth at home. The majority (94.7%) of women delivered their baby. Only 23.5%, of women got counseling about obstetric fistula once upon a time. In addition, 41.4% of women participated in a monthly regular pregnant women’s conference led by health extension workers (Table 2).

**Table 2:** Obstetrics characteristics of women of reproductive age in Banja District, Awi zone, Northwestern Ethiopia, July 2021(n = 773)

Variables	Category	n	%
Number of pregnancies	Nulligravida	145	18.8
	Primigravida	57	7.3
	Multigravida	244	31.6
	Grand multigravida	327	42.3
Number of deliveries	Nulliparous	148	19.1
	Primiparous	61	7.9
	Multiparous	564	73.0
History of abortion	Yes	129	16.7
	No	644	83.3
History of stillbirth	Yes	71	9.2
	No	702	90.8
ANC follow-up (n = 628)	Yes	316	50.3
	No	312	49.7
Number of antenatal care visit(n = 316)	1 visit	16	5.1
	2–3 visit	190	60.1
	≥ 4 visit	110	34.8
Place of delivery (n = 625)	Health institution	488	78.1
	Home	137	21.9
Reason to delivery at home	Lack of transport	95	66.4
	No nearby facility	37	25.9
	Others*	11	7.7
Mode of delivery (n = 625)	SVD	592	94.7
	Instrumental delivery	15	2.4
	Cesarean section delivery	18	2.9
Postnatal follow-up (n = 625)	Yes	354	56.6
	No	271	43.4
Ever used family planning	Yes	336	43.47
	No	437	56.53
Get counseling about obstetric fistula	Yes	182	23.5
	No	591	76.5
When do you get the counseling?	Prenatal	93	45.4
	Antenatal	100	48.7
	Postnatal	12	5.9
Ever participated in a pregnant women conference?	Yes	260	33.63
	No	513	66.37
Time take in a minute to reach a health facility	≤ 30 min	248	32.1
	> 30 min	525	67.9

Others*; poor road condition and poor maternal decision-making power, SVD: spontaneous vaginal delivery

## Women’s knowledge about obstetric fistula

**Fig. 2:**
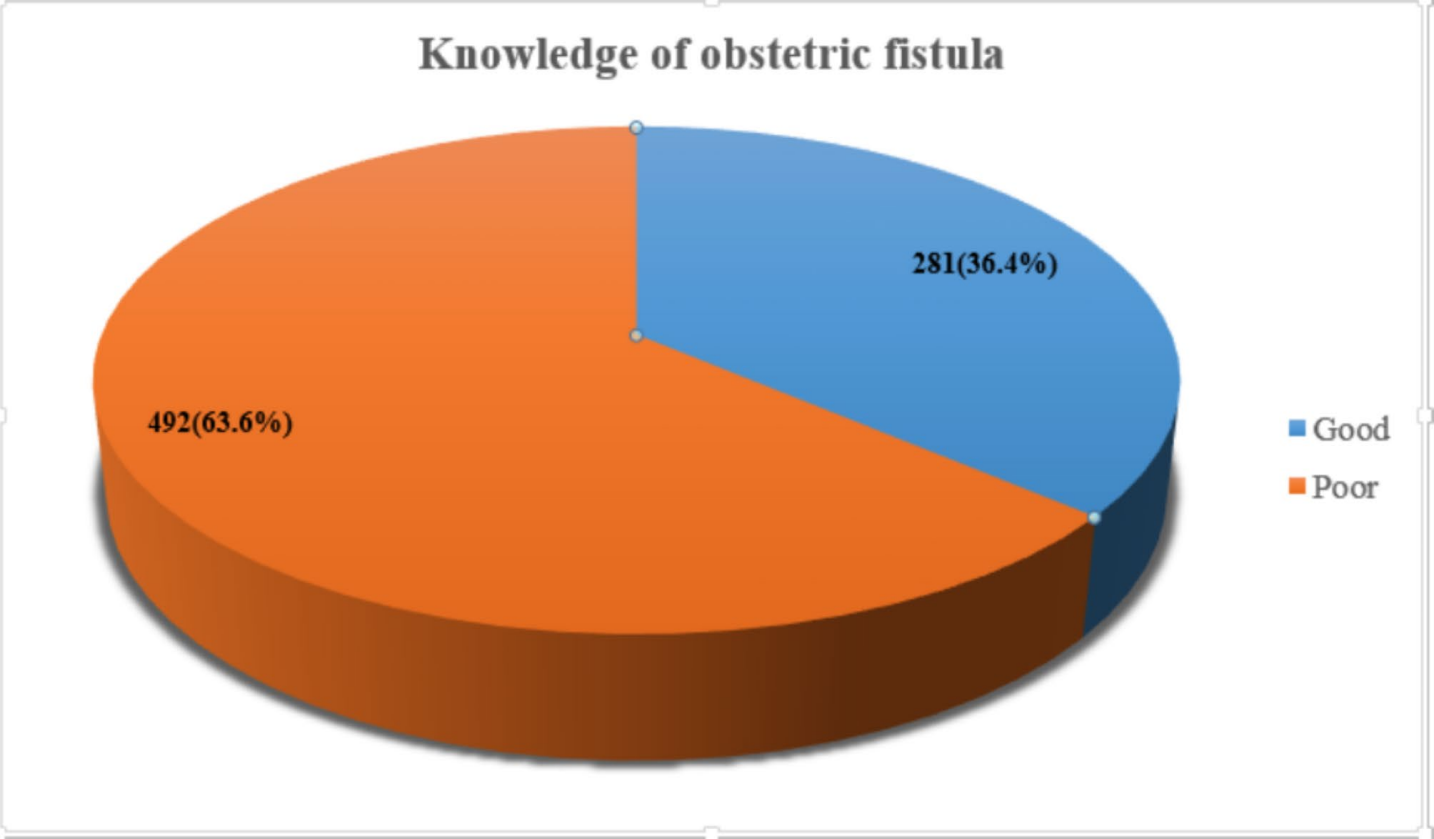
Overall knowledge of obstetric fistula among women in the reproductive age in Banja District, Northwestern Ethiopia, July 2021(n = 773)

Out of 773 study participants, the overall women’s knowledge about obstetric fistula in the study was 281 (36.4% (95% CI: 32.9-39.7%) (Fig. 2). The most frequently cited sign and symptoms of obstetric fistula were urinary incontinency (33.0%) and faecal incontinence (32.7%). Regarding risk factors, prolonged labor (26.9%) and child marriage (26.8%) were frequently mentioned risk factors for obstetric fistula. Delaying the age of first pregnancy (34.4%) cessation of harmful traditional practices like female genital mutilation (30.4%) were frequently mentioned prevention methods of obstetric fistula by the study participants (Table 3).

**Table 3:** Knowledge about obstetric fistula among women in Banja District, Northwestern Ethiopia, July 2021(n = 773)

Variables	Category	n	%
Ever heard of obstetric fistula?	Yes	299	38.7
	No	474	61.3
Source of information	Health professional	198	25.6
	School	132	17.1
	Mass media (radio, TV)	43	5.6
	Family/friend	37	4.8
	Others*	12	1.6
Know the type of fistula?	Yes	105	13.6
	No	668	86.4
Type of obstetric fistula	Recto-Vaginal Fistula (RVF)	4	3.8
	Vesico-Vaginal Fistula (VVF)	6	5.7
	Both	95	90.5
Know the sign/symptoms of obstetric fistula?	Yes	256	33.1
	No	517	66.9
Know sign/symptoms of obstetric fistula	Urinary incontinency	255	33.0
	Faecal incontinency	253	32.7
	Vulvar irritation	86	11.1
	Foul-smelling vaginal discharge	172	22.3
	Leakage of gas/faeces into the vagina	79	10.2
	Pain while having sex	17	2.2
Know the causes/risk factors of obstetric fistula?	Yes	228	29.5
	No	545	70.5
Causes/risk factors of obstetric fistula	Prolonged labor	208	26.9
	Obstructed labor	151	19.5
	Childhood malnutrition	30	3.9
	Operative delivery	17	2.2
	Early marriage	207	26.8
	Younger age	9	1.2
	Home delivery	163	21.1
	Unspaced childbirth	32	4.1
	Lack of obstetrics care	80	10.3
Is obstetric fistula preventable?	Yes	291	37.6
	No	482	62.4
Prevention methods of obstetric fistula	Delaying the age of first pregnancy	266	34.4
	Cessation of harmful traditional practices	235	30.4
	Timely visit/seeking of skilled obstetric care	223	28.8
	Avoiding poverty	149	19.3
	Empowering women and female education	191	24.7
	Skilled care at birth	188	24.3
	Family planning use	119	15.4
Is obstetric fistula a treatable condition?	Yes	325	42.0
	No	448	58.0
Type of treatment mentioned	Medical treatment	317	39.5
Overall knowledge about obstetric fistula	Good knowledge	281	36.4
	Poor knowledge	492	63.6

Others*: fistula victim, community meeting, TV (television)

## Factors associated with women’s knowledge about obstetrics fistula

Ten variables (women’s level of education, women’s occupation, getting counselling about obstetric fistula, history of abortion, history of stillbirth, postnatal follow-up, participated in pregnant women conference, ANC follow-up, residence and having TV/radio) were variables showing significant association at a p-value of ≤ 0.25. After controlling the confounding variables, this study identified six independent factors affecting the women’s’ knowledge level of the obstetric fistula. These were women’s level of education, women’s occupation, getting counseling about obstetric fistula, participating in pregnant women conferences, ANC follow-up, residence, and having TV/radio. Women who had completed primary education (AOR: 3.47, 95%CI: 2.01–5.98) and secondary education and above (AOR: 3.30, 95%CI: 1.88–5.80) were 3 times more likely to have good knowledge about obstetric fistula than women who unable to read and write. The odds of knowledge about obstetric fistula were about 6.78 times higher among those study participants who were a student as compared to farmers (AOR: 6.78, 95%CI: 3.88–11.86). Similarly, the odds of knowledge about obstetric fistula were higher among participants who get counseling about obstetric fistula as compared to their counterparts (AOR: 6.22, 95%CI: 3.78–10.24). Women who participated in pregnant women’s conferences were 3.36 times more likely to be knowledgeable about obstetric fistula than those who have not participated at pregnant women conferences (AOR: 3.36, 95%CI: 1.99–5.66). Women who had ANC follow-up history at time of pregnancy so far were 2.40 times more likely to be knowledgeable about obstetric fistula as compared to their counterparts (AOR: 2.40, 95% CI: 1.39–4.13). Those women who were urban dwellers were 3.19 times more likely knowledgeable than rural dwellers (AOR: 3.19, 95% CI: 1.33–7.66). Women who have access to mass media (TV/radio program) had also a higher odds of knowledge level about obstetric fistula as compared to their counterpart (AOR: 1.68, 95% CI: 1.10–2.60) (Table 4).

**Table 4:** Factors associated with knowledge of obstetric fistula among women in Banja District, Northwestern Ethiopia, July 2021(n = 773)

Variables	Knowledge of obstetric fistula		COR (95%CI)	AOR (95%CI)
	Good (%)	Poor (%)		
Women’s level of education				
Can’t read and write	94(23.2)	311(76.8)	1	1
Can read and write	35(31.5)	76(68.5)	1.52(0.96–2.42)	0.97(0.54–1.75)
Primary education	67(54.5)	56(45.5)	3.96(2.59–6.04)	3.47(2.01–5.98)*
Secondary education and above	85(63.4)	49(36.6)	5.74(3.77–8.74)	3.30(1.88–5.80)*
Women’s occupation				
Government employee	17(77.3)	5(22.7)	8.46(3.07–23.36)	1.78(0.47–6.73)
Student	85(61.2)	54(38.8)	3.92(2.65–5.79)	6.78(3.88–11.86)*
Private employee	12(25.0)	36(75.0)	0.83(0.42–1.64)	0.33(0.13–1.82)
Housewife	20(39.2)	31(60.8)	1.61(0.89–2.91)	0.51(0.19–1.37)
Farmer	147(28.7)	366(71.3)	1	1
Getting counselling about obstetric fistula				
Yes	133(73.1)	49(26.9)	8.12(5.57–11.84)	6.22(3.78–10.24)*
No	148(25.0)	443(75.0)	1	1
History of abortion				
Yes	43(33.9)	84(66.1)	0.87(0.59–1.31)	1.47(0.85–2.54)
No	238(36.8)	408(63.2)	1	1
History of stillbirth				
Yes	18(25.4)	53(74.6)	0.56(0.32–0.99)	0.54(0.24–1.22)
No	263(37.5)	439(62.5)	1	1
Postnatal follow-up				
Yes	157(44.4)	197(55.6)	1.89(1.41–2.55)	0.63(0.34–1.16)
No	124(29.6)	295(70.4)	1	1
Participated in pregnant women conference				
Yes	146(56.2)	114(43.8)	3.58(2.62–4.91)	3.36(1.99–5.66)*
No	135(26.3)	378(73.7)	1	1
ANC follow-up				
Yes	163(51.6)	153(48.4)	3.06(2.26–4.15)	2.40(1.39–4.13)*
No	118(25.8)	339(74.2)	1	1
Residence				
Urban	44(75.9)	14(24.1)	6.34(3.41–11.80)	3.19(1.33–7.66)*
Rural	237(33.1)	478(66.9)	1	1
Having TV/radio				
Yes	120(51.3)	114(48.7)	2.47(1.80–3.39)	1.68(1.10–2.60)*
No	161(29.9)	378(70.1)	1	1

* Statistically significant at a *p-*value of less than 0.05

## Discussion

The present study determined the level of women’s knowledge on obstetric fistula and its associated factors in northwestern Ethiopia. Only one-in-three women, were found to be knowledgeable about obstetric fistula in the study area. Since the prevalence of knowledge of obstetric fistula in this study is low, this initiates health workers to focus in giving of health education, especially in obstetric fistula to increase the knowledge of women and for researchers to do researches by using longitudinal and qualitative study designs to identify why knowledge of obstetric fistula is too low.

This study finding on the prevalence of women’s knowledge about obstetric fistula is in line with the study conducted in Burkina Faso (36.4%) [21]. However, the present study finding is higher than studies reported from Ghana (29%) [12], and Cameroon (23.2%) [31]. This difference might be attributed to variation in the study nature, sociodemographic characteristics of participants of the study, and differences in sample size, for instance, small study participants were enrolled in the study reported from Ghana. In contrary, the finding of this study is lower than the studies done in Nigeria (57.8%) [32], and in Ethiopia (40.8–41.2%) [18][33]. This is because now a days health education about the health status of the community including obstetric fistula is given by health providers and also by media.

In this study, education and occupation are the sociodemographic characteristics of women which are significantly associated with knowledge of obstetric fistula. Accordingly, women who attended primary education, and secondary education and above were 3.47 and 3.30 times more likely knowledgeable about obstetric fistula as compared to women who cannot read and write. This finding is consistent with previous studies reported from Ghana [12], Burkina Faso [21], and another study in Ethiopia [18][33]. Obviously education is crucial for awareness and contributes to women empowerment to minimize risk of obstetric fistula, mainly child marriage, promotes gender equality [34, 35][36].

Consistent with the previous study [37], the occupation of the women is significantly associated with the women’s knowledge level about obstetric fistula. In doing so, the odds of being knowledgeable about obstetric fistula were 6.78 times more common among participants who are students by their occupation compared to those who are farmers. Similarly, the finding of this study noted that women who had ever participated in pregnant women conferences were 3.36 times more likely knowledgeable about obstetric fistula as compared to their counterparts. The finding of this study bears similarity with a study conducted in Ethiopia [38]. This is due to the fact that the pregnant women’s discussion forum is one of the widely recognized platforms, where health personnel provide health education about maternal health.

According to this study, the knowledge level of obstetric fistula is significantly associated with counseling about obstetric fistula. Women who get counseling about obstetric fistula were about six times more likely knowledgeable about obstetric fistula as compared to their counterparts. This is explained by receiving counseling services about obstetric fistula increase in women’s knowledge about obstetric fistula and fistula prevention, speaks to the benefit of having one-on-one counseling with a trained individual during which perceptions and misconceptions can be addressed [39]. According to this study, the knowledge level of obstetric fistula is higher in urban areas (75.9% in urban vs. 33.4% in rural). This finding is in line with the study conducted in Burkina Faso [21]. This might be due to being an urban resident would offer a chance to access information about health and health-related issues including awareness about obstetric fistula, as most of the health facilities are confined to urban areas. On the other hand, women living in rural areas could have lower access and exposure to mass media which might further reduce their level of awareness and knowledge on health-related issues [40]. It is important to note that mass media such as TV, radio, and newsletters have become a significant source for raising awareness of the community about health and health-related issues including obstetric fistula [41].

Having antenatal care follow-up is another determinant factor for knowledge of obstetric fistula among women of reproductive age. Accordingly, the odds of knowledge about obstetric fistula were 2.4 times more common among women who have antenatal care follow-up as compared to those who haven’t antenatal care follow-up. This finding is in congruent with the study conducted in India [42], Ghana [12], and Ethiopia [38, 43].

## Strength and limitation of the study

This study was conducted at the community-based house-to-house level, comprised of both urban and rural resident participants, applied probability sampling method with a scientifically sound approach for sample size determination for generalization can be taken as strength of the study. Despite this strength, the cause-and-effect relationship may be affected by temporality issues due to the nature of the cross-sectional study design and the response of participants might be affected by recall bias.

## Conclusion

In this study, the overall knowledge of obstetric fistula among reproductive-age women was unacceptably low. Women’s level of education, women’s occupation, getting counselling about obstetric fistula, participated in pregnant women conferences, having antenatal care follow-up, being from an urban resident, and having TV/radio were variables that have a significant association with knowledge of women’s about obstetric fistula. Therefore, empowering women in education, promoting antenatal care, and reinforcing pregnant women’s counseling conference platforms could substantially optimize women’s knowledge of obstetric fistula. Concerned key stakeholders, such as healthcare workers, mainly health extension workers and local leaders should work on communities’ awareness creation about of obstetric fistula, and its risk factors in the study area and other low-income countries to save lives of women and improve their quality of life. In addition, research should ascertain further why the prevalence of women’s knowledge about obstetric fistula is low using mixed method with extended time and intervention.

## Data Availability

The participants de-identified data used for current study will be available upon submitting reasonable request from the corresponding author (MT) in either SPSS or Stata format and as per the permission obtained from senior project principals (AS, TA).

## Declarations

### Computing interests

All authors have declared no computing interest.

## Abbreviations

ANC: Antenatal Care.
IHRERC: Institutional Health Research Ethics Review Committee.
RVF: Recto Vaginal Fistula.
SDGs: Sustainable Development Goals.
SPSS: Statistical Package for Social Sciences.
VVF: Vesico Vaginal Fistula.
SNNPR: Southern Nation Nationalities and Peoples Region.
